# A newly discovered sRNA is involved in the virulence regulation of *Salmonella pullorum*

**DOI:** 10.3389/fvets.2025.1651294

**Published:** 2025-09-22

**Authors:** Ting He, Meiling Huang, Yuling Sun, Yonghui Ding, Nanlong Zhou, Meihong Fu, Tiansen Li

**Affiliations:** ^1^School of Tropical Agriculture and Forestry, Hainan University, Haikou, China; ^2^School of Pharmacy, Hainan Medical University, Haikou, China; ^3^Department of Basic Medicine, Hainan Vocational University of Science and Technology, Haikou, China

**Keywords:** *Salmonella pullorum*, sRNA, HD11, infection, virulence regulation

## Abstract

**Background:**

*Salmonella pullorum*, the primary pathogen responsible for avian pullorum disease, has imposed substantial economic losses on the poultry industry. sRNAs, a class of small non-coding RNAs, have been identified in numerous bacterial species and serve as pivotal regulatory factors in bacteria.

**Methods:**

A bacterial infection assay was conducted to detect the differential transcription levels of sRNA12 in the macrophage cell HD11. Environmental stress tests, intracellular survival assays, target gene transcription analyses and chick virulence tests were conducted to compare the wild-type strain and the ΔsRNA12 deletion strain.

**Results:**

A significant 7.5-fold increase in the transcription level of sRNA12 was observed during the invasion of host cells by bacteria. Under hyperosmotic conditions, the survival ability of the deletion strain was markedly reduced, while in a highly oxidative environment, it was significantly enhanced. Compared with the wild-type strain, the colonization ability of the ΔsRNA12 deletion strain in HD11 cells was enhanced by 3.5-fold. The transcription levels of most target genes of sRNA12, such as *ompD*, *siiE*, and *prgH*, were significantly upregulated. The LD_50_ of the deletion strain in chicks was approximately three times lower than that of the wild-type strain. Moreover, the colonization abilities of the deletion strain in the liver, spleen, and cecum of chicks were significantly enhanced and it induced more severe organ lesions.

**Conclusion:**

The findings suggest that the deletion of sRNA12 enhances the virulence of *S. pullorum*. This research provides novel insights into elucidating the pathogenic mechanism of *S. pullorum* and the associated regulatory signaling pathways.

## 1 Introduction

*Salmonella*, a facultative anaerobic bacterium belonging to the family Enterobacteriaceae, is a Gram-negative bacterium. Currently, approximately 2,600 serotypes of *Salmonella* have been identified worldwide (1–3). Most of them possess flagella, with the exceptions of *Salmonella gallinarum* and *S. pullorum*. *S*. *pullorum* predominantly infects chicks within 2 weeks of age, leading to frequent disease occurrences. In severe cases, the mortality rate can reach up to 100%. Moreover, broiler chickens are more susceptible to *S*. *pullorum* infection compared to layer chickens (4). Adult chickens can also be infected by *S*. *pullorum*. Usually, the infection follows a chronic course, and the bacterium can be directly transmitted into eggs through the reproductive system, vertically spreading to the chicks (5). The presence of *Salmonella* poses a serious threat to food safety and the healthy development of the livestock industry (6, 7).

Small non-coding RNA (sRNA) can be transcribed but generally does not code for proteins. After transcription, it can form stable secondary structures and is widely present in bacteria. Currently, the known sRNAs mainly exert their functions by completely or incompletely matching with the mRNA of target genes, affecting the transcription and translation processes of these target genes. Most of the sRNAs identified in *Salmonella* block translation by directly binding to the ribosome binding site (RBS) in the 5′-untranslated region (5’-UTR) of the target mRNA. For instance, GcvB blocks the translation of oppA and dppA mRNAs (8), and MicC blocks the translation of hilD mRNA (9). Additionally, sRNAs can also inhibit the initiation of translation by binding to the coding sequence (CDS) region of the target mRNA. For example, SdsR inhibits the translation initiation of ompD mRNA (10), and PinT inhibits the translation initiation of hilA mRNA (11). Conversely, some sRNAs promote translation by binding to the 5’-UTR of the target mRNA. These target mRNAs contain intrinsic secondary structures in their 5’-UTR that inhibit ribosome binding. When an sRNA binds to this inhibitory sequence, the RBS can then bind to the ribosome, facilitating the initiation of translation (12). An example is that RyhB-1 and/or RyhB-2 promote the expression of sipA (13). sRNAs can also mediate the decay of target mRNAs. An sRNA binds to the target mRNA and recruits the endoribonuclease RNase E. RNase E reaches and cleaves the target mRNA in a linear extension manner at the 3′ end of the duplex region (14). For example, the base pairing between DsrA and pflB mRNA recruits RNase E to degrade pflB mRNA (15).

sRNAs are crucial regulatory factors in bacteria and play key roles in various aspects such as regulating biofilm formation, environmental adaptation, iron homeostasis, bacterial metabolism, and virulence (16). sRNAs like GcvB, RyhB, DsrA, and ArcZ can participate in regulating the stress response, enabling bacteria to adapt to environmental changes (17–23). sRNAs such as SaaS and ArcZ are involved in the regulation of bacterial biofilm formation (24, 25). sRNAs including SdsR, Spot42, CsrB/CsrC, MicC, RyhB, and PinT can regulate bacterial virulence (9, 26–29). MicC can regulate the expression of outer membrane proteins (30), and RyhB can regulate iron uptake and utilization (31). These sRNAs interact with multiple targets, enabling *Salmonella* to form a complex regulatory molecular network that collectively influences the growth and virulence of the bacteria.

In a previous study, we employed RNA-Seq to predict the presence of 110 novel sRNAs in *S*. *pullorum*. Based on the number of reads and bioinformatics analysis, we selected a representative sRNA, sRNA12, which is hypothesized to play a crucial role in the bacteria-host interaction process. Therefore, we selected sRNA12, constructed the ΔsRNA12 deletion strain, and explored the virulence-related functions of both the wild-type strain and the ΔsRNA12 deletion strain. The aim of this study is to elucidate the role that sRNA12 plays in the regulation of bacterial virulence.

## 2 Materials and methods

### 2.1 Bacterial strains, plasmids, and primers

The *S*. *pullorum* (ATCC10398) used in this study was preserved in our laboratory. The ΔsRNA12 gene deletion strain was constructed by the *λ*-Red homologous recombination method (32). The plasmids pKD20, pKD4, and pCP20 used in the construction process were preserved in our laboratory. The primers used in the experiment are shown in Table 1.

**Table 1:** The primers used in the experiment.

Primer name	Primer sequence (5′ → 3′)	Product length (bp)
sRNA12-F	GCAGGCGTCAAAACAGGT	344
sRNA12-R	CACTTTGTAAAAAGCATCCTG	
qpsRNA12-F	GGGGTGCTTCACTCAACG	131
qpsRNA12-R	CTCTTATGTTCGCCTTCTGG	
16 s rRNA-F	CAGAAGAAGCACCGGCTAACTC	87
16 s rRNA-R	GCGCTTTACGCCCAGTAATT	
up-cm-down-F	CTACACAAAATCATTCGGGATGCATAAAAAAACGCCTGCCCGGTTAAGGGAGCGATTGTGTAGGCTGGAG	1,152
up-cm-down-R	CAGTCTTCATTTGTCGCTATTCGGTGACGAATAATCTATTGATTTATAATTTAACGGCTGACATGGGAATTAG	
sRNA12-out-F	CGCAGTAATTCCCTCTTGCC	542/1250/320
sRNA12-out-R	AACCCCTTATCCCGCACG	
bapA-F	AACGACAATACCCCCACTCTG	199
bapA-R	CTGATTCGCTACTTTCGCCTT	
ompC-F	CCGATAACTGGGAAATGGTAGG	237
ompC-R	CCACGGTGTTGTCTGTATTAGG	
sseC-F	TACGAAAGAGGGGGTGAAAG	188
sseC-R	CACATCTGATAGCCTGTAAGCC	
ompD-F	AACCAGGTGAAAGCAGCGAG	241
ompD-R	GGTAAGCGATGGACGGACG	
siiE-F	TTACAAATAGCACCCTGCCAAC	102
siiE-R	TAGCAACAATGACCTCACCAAG	
sseG-F	ACAGTGCTAAGTGGTGGAGGC	95
sseG-R	TGATGGTAGATAAGACAGGCGAC	
prgH-F	CAACACCTGAAAACGCTCCT	200
prgH-R	CCGAGCAGCCTGAGAAGTT	
sseJ-F	TCAATACTTTGGCGGAAGGTTT	190
sseJ-R	AGAAGGGGTGTAAGATGCGACT	
fimW-F	ATTGATCTTACATAAGCGAGCG	153
fimW-F	AGTGAAAGTAAAGCACCCGTT	
rho-F	TAGAACCGTTACCACGCTCC	206
rho-R	AATCCGCCGTTTCAACCTCC	
16 s rRNA-F	CAGAAGAAGCACCGGCTAACTC	87
16 s rRNA-R	GCGCTTTACGCCCAGTAATT	

The underlined part of the primers for the targeting fragment is the primer fragment of the chloramphenicol resistance gene.

### 2.2 Verification of sRNA12 and detection of its transcription level

*S*. *pullorum* was revived and cultured in LB medium. Total RNA was extracted from bacterial cells using Trizol reagent (Thermo Fisher, MA, USA, Cat No. 16096020), and a reverse transcription kit (Takara, Shiga, Japan, Cat No. RR037A) was used to reverse-transcribe it into cDNA. The full-length sequence of sRNA12 was amplified by PCR using the primers sRNA12-F and sRNA12-R listed in Table 1. Chicken macrophage HD11 cells were cultured in DMEM medium (Cytiva, MA, USA, Cat No. SH30243.01) supplemented with 10% fetal bovine serum (FBS; Gibco, MA, USA, Cat No. 10099-141C) and 50 μg/mL of penicillin–streptomycin antibiotics (Servicebio, Wuhan, China, Cat No. G4015). The cells were cultivated in an incubator at 37°C with 5% CO_2_ until the confluence reached approximately 90%. 1.5 h before the bacterial infection of the cells, the cell culture medium was replaced with DMEM medium containing 10% FBS without antibiotics. *S*. *pullorum* was added to the cells in a 6-well plate at a multiplicity of infection (MOI) ratio of 100:1, and then the plate was placed back into the incubator at 37°C with 5% CO_2_ for further incubation for 1 h, and this time point was recorded as 0 h. The cells were washed three times with sterile PBS, and then DMEM medium containing 10% FBS and 100 μg/mL of gentamicin (Solarbio, Beijing, China, Cat No. G8170) was added. The cells were continuously incubated at 37°C for another 1 h. After that, the cells were washed three times with sterile PBS, and DMEM medium containing 10% FBS and 10 μg/mL of gentamicin was added. Starting from the time when the medium was replaced with the one containing a lower concentration of gentamicin, the cells were collected at 2 h, 4 h, 6 h, 8 h, and 10 h after the bacteria survived intracellularly. *S*. *pullorum* that did not infect the cells was used as a control (named sp), and 16 s rRNA was used as an internal reference. With qpsRNA12-F, qpsRNA12-R, 16 s rRNA-F, and 16 s rRNA-R (Table 1) as primers, the transcription level of sRNA12 was detected by quantitative real-time PCR (qRT-PCR).

### 2.3 Construction of the ΔsRNA12 deletion strain

The ΔsRNA12 deletion strain of *S*. *pullorum* was constructed using the *λ*-Red homologous recombination method (32). Briefly, 50-bp sequences flanking the sRNA12 gene were selected as the upstream and downstream homologous recombination arms for gene targeting. These arms were ligated to both sides of the specific primers for the chloramphenicol resistance gene. Using up-cm-down-F and up-cm-down-R as primers (Table 1), the chloramphenicol resistance gene was amplified from the pKD3 plasmid by PCR to obtain the homologous recombination fragment. Electrocompetent cells of *S*. *pullorum* were prepared. The homologous recombination plasmid pKD20 and the homologous recombination fragment were electrotransformed into the competent cells, respectively. Expression of the recombinant proteins was induced with 0.5% L-arabinose (Yeasen, Shanghai, China, Cat No. 60312ES10). Positive colonies were screened based on chloramphenicol resistance. The deletion of sRNA12 was verified using sRNA12-out-F and sRNA12-out-R as outer primers.

The pCP20 plasmid was electrotransformed into the competent cells of the ΔsRNA12 deletion strain. Positive colonies were screened based on chloramphenicol and ampicillin resistance. These colonies were then cultured overnight in antibiotic-free LB liquid medium at 42°C and 180 r/min to eliminate the temperature-sensitive pCP20 plasmid. The elimination of the chloramphenicol resistance marker was verified using sRNA12-out-F and sRNA12-out-R as outer primers (Table 1).

### 2.4 Identification of the biological characteristics of the ΔsRNA12 deletion strain

To evaluate the growth characteristics of the ΔsRNA12 deletion strain, we investigated the growth kinetics of both the wild-type strain and the ΔsRNA12 deletion strain (32). Both the *S*. *pullorum* wild-type strain and the ΔsRNA12 deletion strain were cultured at 37°C with a shaking speed of 180 rpm. The absorbance values at 600 nm (OD_600 nm_) were recorded every 2 h for a total of 24 h using a spectrophotometer (Metash, Shanghai, China, Model UV-5500).

### 2.5 *In vitro* environmental stress detection of bacteria

To analyze the role of sRNA12 in resisting and adapting to the host environment, *in vitro* LB media with acidic (pH = 4), hyperosmotic (10% NaCl), and highly oxidative (2 mM H_2_O_2_) conditions were set up to mimic the environmental stimuli within the host and macrophages (33). The wild-type strain and the ΔsRNA12 deletion strain were cultured in the above LB media at 37°C and 180 r/min for 1.5 h, then serially diluted and spread on LB agar plates, and cultured overnight at 37°C to detect the bacterial survival rate.

### 2.6 Detection of bacterial invasion ability into cells

The cell infection procedure was the same as that described in 2.2. The wild-type strain and the ΔsRNA12 deletion strain of *S*. *pullorum* were added to the cells in 6-well plates at a multiplicity of infection (MOI) of 100:1, respectively. At 0 h, 6 h, 12 h, and 24 h, 0.2% Triton X-100 (Biotopped, Beijing, China, Cat. No. T6200G) was added to lyse the cells, and the samples were collected. The samples were evenly spread on LB agar plates and cultured overnight at 37°C. The colony-forming units (CFUs) at each time point were calculated (33).

### 2.7 Prediction of sRNA12 target genes and detection of target gene levels during infection

We used the IntaRNA 3.3.2 software to predict the target genes of sRNA12 (33). Based on the ranking of the lowest free energy, the top 10 target genes related to bacterial virulence were selected for the detection of relative transcription levels. At 0 h, 4 h, 8 h, and 12 h after the bacteria infected the cells, 0.2% Triton X-100 was added to lyse the cells, and the samples were collected. Total RNA was extracted and reverse-transcribed. Real-time fluorescent quantitative PCR detection was performed using the primers listed in Table 1.

### 2.8 Evaluation of the virulence of the wild-type strain and the ΔsRNA12 deletion strain in chicks

All our animal experiments were conducted in accordance with the protocols approved by the Animal Protection and Ethics Committee of Hainan University (Haikou, China). To evaluate the virulence of the wild-type strain and the ΔsRNA12 deletion strain of *S. pullorum* in chicks, 70 three-day-old healthy Wenchang chickens were randomly divided into 7 groups, with 10 chickens in each group. Each group was orally administered with the wild-type strain and the ΔsRNA12 deletion strain at three concentration gradients of 2 × 10^8^, 2 × 10^9^, and 2 × 10^1^⁰ CFU/500 μL, respectively. Ten chicks in the control group were orally administered with 500 μL of sterile PBS. The mortality rate of each group was recorded within 14 days after challenge, and the median lethal dose (LD_50_) of the two strains was determined using the modified Karber method (33).

### 2.9 Detection of organ colonization ability of the wild-type strain and the ΔsRNA12 deletion strain

Sixty healthy Wenchang chickens were randomly divided into three groups, with 20 chickens in each group. Chicks were orally infected with the wild-type strain and the ΔsRNA12 deletion strain at a dose of 3 × 10^9^ CFU/500 μL, respectively, while chicks in the control group were orally administered with an equal volume of sterile PBS. On days 1, 3, 5, 7, and 9 post-infection, the chicks were humanely euthanized. The liver, spleen, and cecum were aseptically collected and homogenized in sterile PBS. After diluting the homogenates, they were inoculated on SS medium (Landbridge, Beijing, China, Cat. No. CM206) and cultured at 37°C for 16 h, and the CFU of each group was calculated.

### 2.10 Histopathological examination

Three chicks from each group that were infected on the 5th day in section 2.9 were humanely euthanized. Their livers, spleens, and ceca were excised and immediately fixed in a 4% paraformaldehyde fixative (Biosharp, Hefei, China, Cat. No. BL539A). After fixation for 24 h, the tissue samples were embedded in paraffin, sectioned, stained with hematoxylin and eosin (H&E), and then subjected to histopathological evaluation under an optical microscope.

### 2.11 Data analysis

Data analysis was performed using GraphPad Prism 8 software. The data were expressed as mean ± standard deviation. Within each group, an independent samples t-test was applied. For repeated measurement data, repeated measures analysis of variance (ANOVA) was used to compare the means among groups (*p < 0.05; **p < 0.01; ***p < 0.001).

## 3 Results

### 3.1 The level of sRNA12 increases significantly after *S. pullorum* invades cells

**Figure 1:**
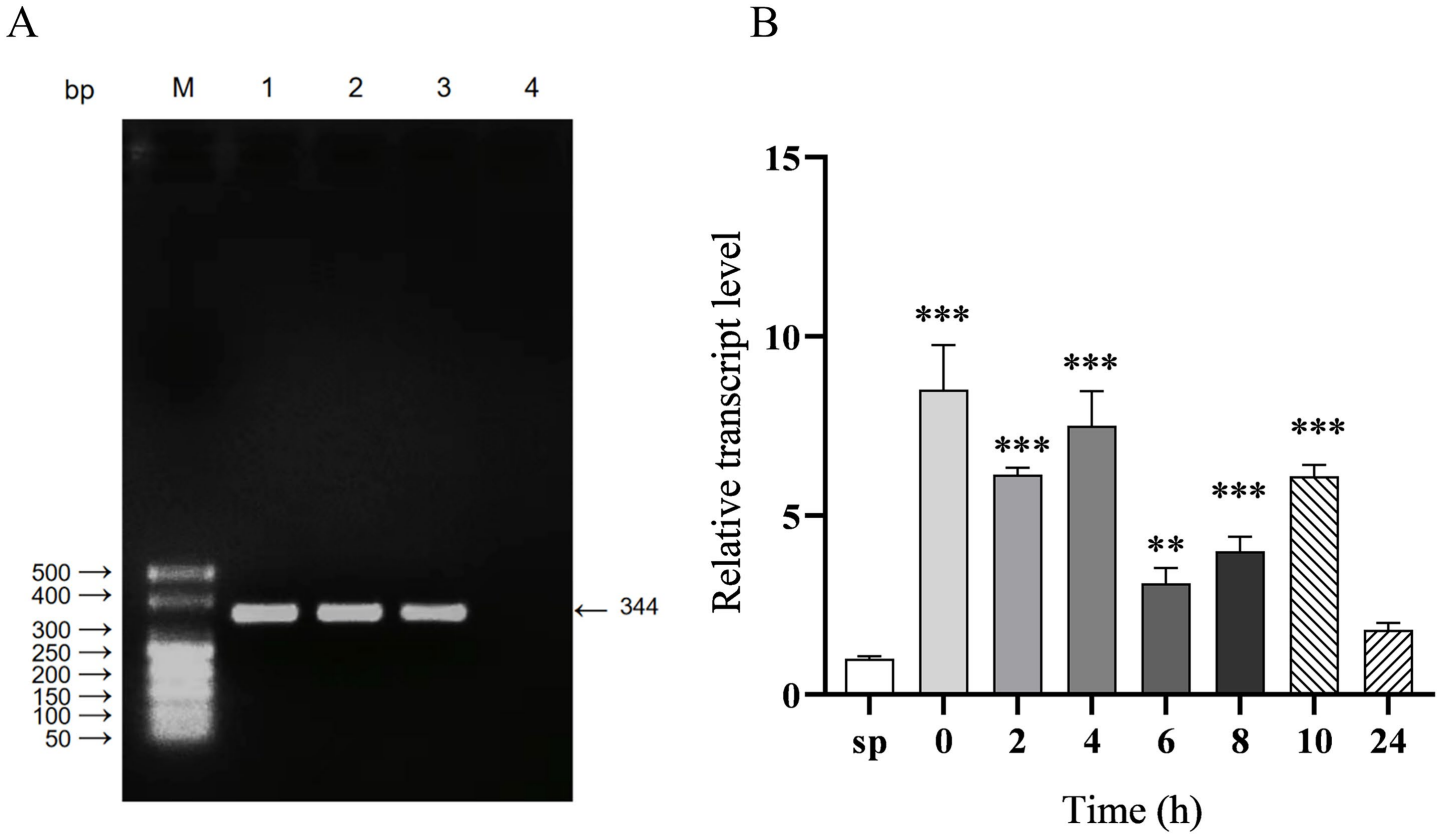
Amplification of sRNA12 and detection of its intracellular transcription level. **(A)** Amplification of sRNA12. Lane M: DL500 DNA Marker; Lane 1–3: sRNA12 of *S. pullorum*; Lane 4: negative control (Supplementary Figure S1); **(B)** Transcript levels of sRNA12. Compared with control group sp., *p < 0.05, **p < 0.01, ***p < 0.001.

The full-length sequence of sRNA12, which is 344 nt in length, was amplified by RT-PCR (Figure 1A). After the bacteria infected HD11 cells, the changes in the transcription level of sRNA12 were detected by qRT-PCR. It was found that compared with the control group, at 0, 2, 4, 6, 8, and 10 h after the bacteria invaded the cells, the level of sRNA12 was significantly increased (*p* < 0.001), being 6.1, 7.5, 3.1, 4.0, and 6.1 times that of the control group, respectively. At 24 h, it decreased to 1.8 times, and the difference was not significant (Figure 1B). These results indicate that sRNA12 may play an important role in the process of bacteria-host interaction.

### 3.2 Construction and biological characteristics of the sRNA12 deletion strain

**Figure 2:**
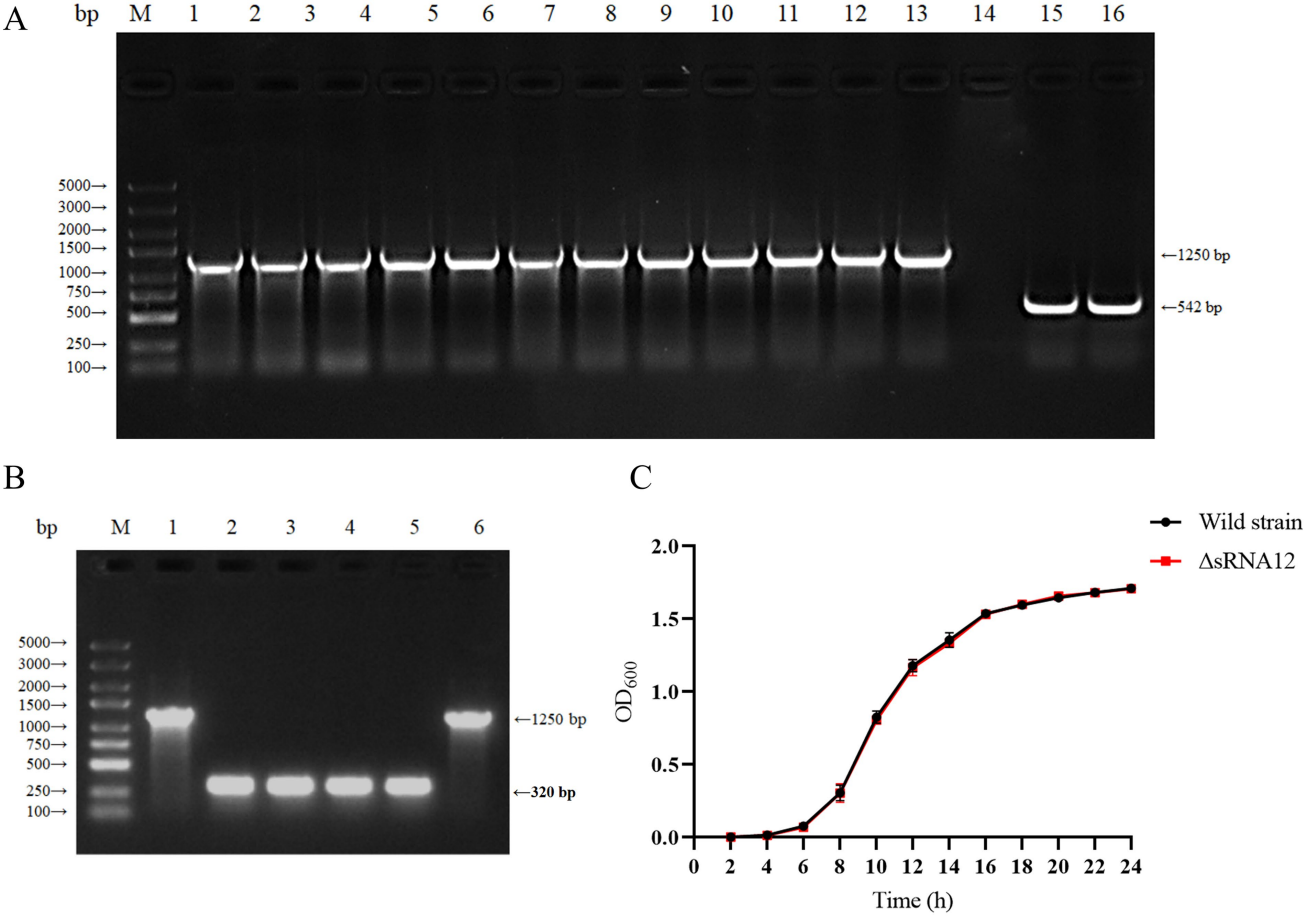
Construction of the sRNA12 deletion strain and determination of its growth rate. **(A)** Identification of homologous recombination of sRNA12. Lane M: DL5000 DNA Marker; Lane 1–13: sRNA12 homologous recombination strain; Lane 14: negative control; Lane 15–16: Wild-type strain of *S. pullorum* (Supplementary Figure S2). **(B)** Identification of sRNA12 knockout strain. Lane M: DL5000 DNA Marker; Lane 1 and 6: sRNA12 homologous recombination strain (Supplementary Figure S3); Lane 2–5: sRNA12 knockout strain. **(C)** Growth curve determination of wild-type and ΔsRNA12 deletion strains.

The ΔsRNA12 deletion strain of *S. pullorum* was generated through homologous recombination (32). The sRNA12 sequence was replaced with the chloramphenicol resistance gene. PCR amplification using the outer primers sRNA12-out-F and sRNA12-out-R yielded a 1,250 bp product. The chloramphenicol resistance gene was removed using the pCP20 plasmid, and a 320 bp product was obtained by PCR amplification. PCR amplification of the wild-type strain of *S. pullorum* produced a 542 bp product. These results confirmed the successful construction of the ΔsRNA12 deletion strain (Figures 2A,B). Growth kinetics data showed that there was no significant difference in the growth rate between the ΔsRNA12 deletion strain and the wild-type strain when cultured in LB medium at 37°C (Figure 2C).

### 3.3 sRNA12 involved in the bacterial response to environmental stress

**Figure 3:**
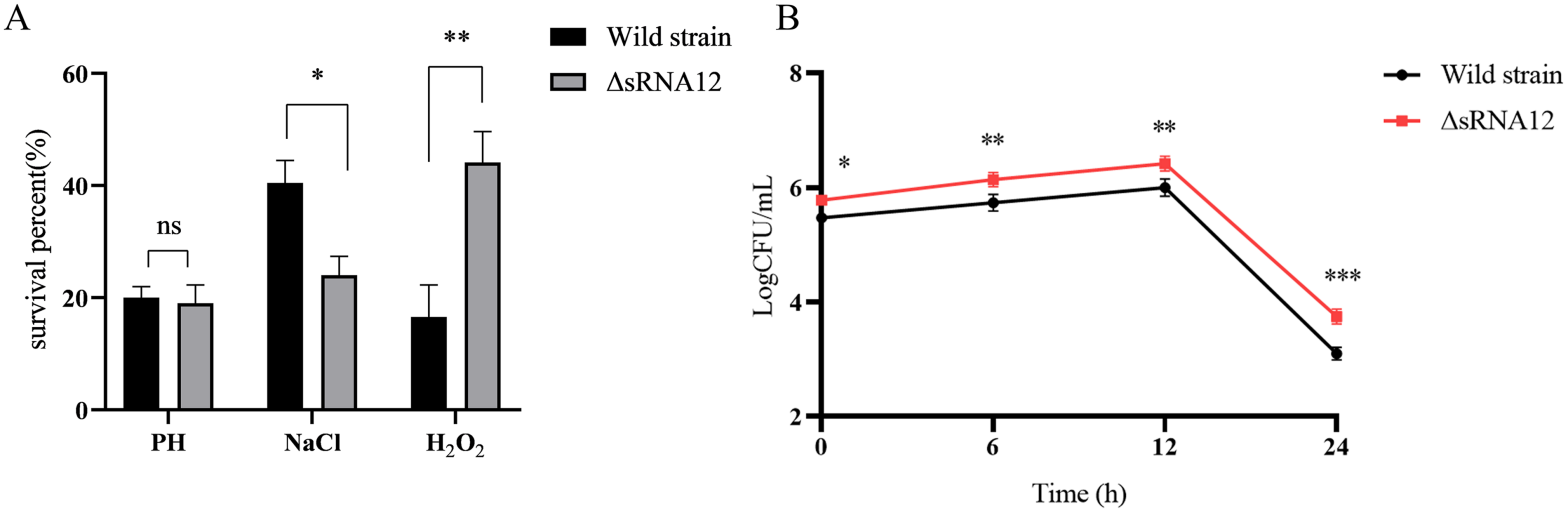
Detection of the environmental stress and intracellular survival of bacteria. **(A)** Survival rates of wild-type strains and ΔsRNA12 deletion strains under different environmental stresses. **(B)** The number of survival of wild-type strains and ΔsRNA12 deletion strains in HD11 cells.

LB media with acidic, hyperosmotic, and highly oxidative conditions were set up *in vitro* to mimic the environmental stimuli within the host and macrophages (33). Compared with the wild-type strain of *S. pullorum*, the survival rate of the ΔsRNA12 deletion strain decreased slightly under acidic conditions, but the difference was not significant. Under hyperosmotic conditions, the survival rate of the wild-type strain was 1.68-fold higher than that of the ΔsRNA12 deletion strain, and the survival rate of the deletion strain decreased significantly (*p* < 0.05). Under highly oxidative conditions, the survival rate of the ΔsRNA12 deletion strain was 2.66 times that of the wild-type strain, and the survival rate of the deletion strain increased significantly (*p* < 0.01) (Figure 3A). These findings indicate that sRNA12 can regulate osmotic pressure and oxidative stress.

### 3.4 The ΔsRNA12 deletion strain enhances bacterial invasion of cells

The wild-type strain and the ΔsRNA12 deletion strain that invaded chicken macrophage HD11 cells were quantified through the gentamicin protection assay. The results showed that at each time point of infection, the bacterial count of the wild-type strain in the cells was significantly lower than that of the ΔsRNA12 deletion strain. This indicates that the deletion of sRNA12 can enhance the ability of bacteria to colonize within cells (Figure 3B).

### 3.5 sRNA12 can affect the transcription of virulence-related target genes

**Figure 4:**
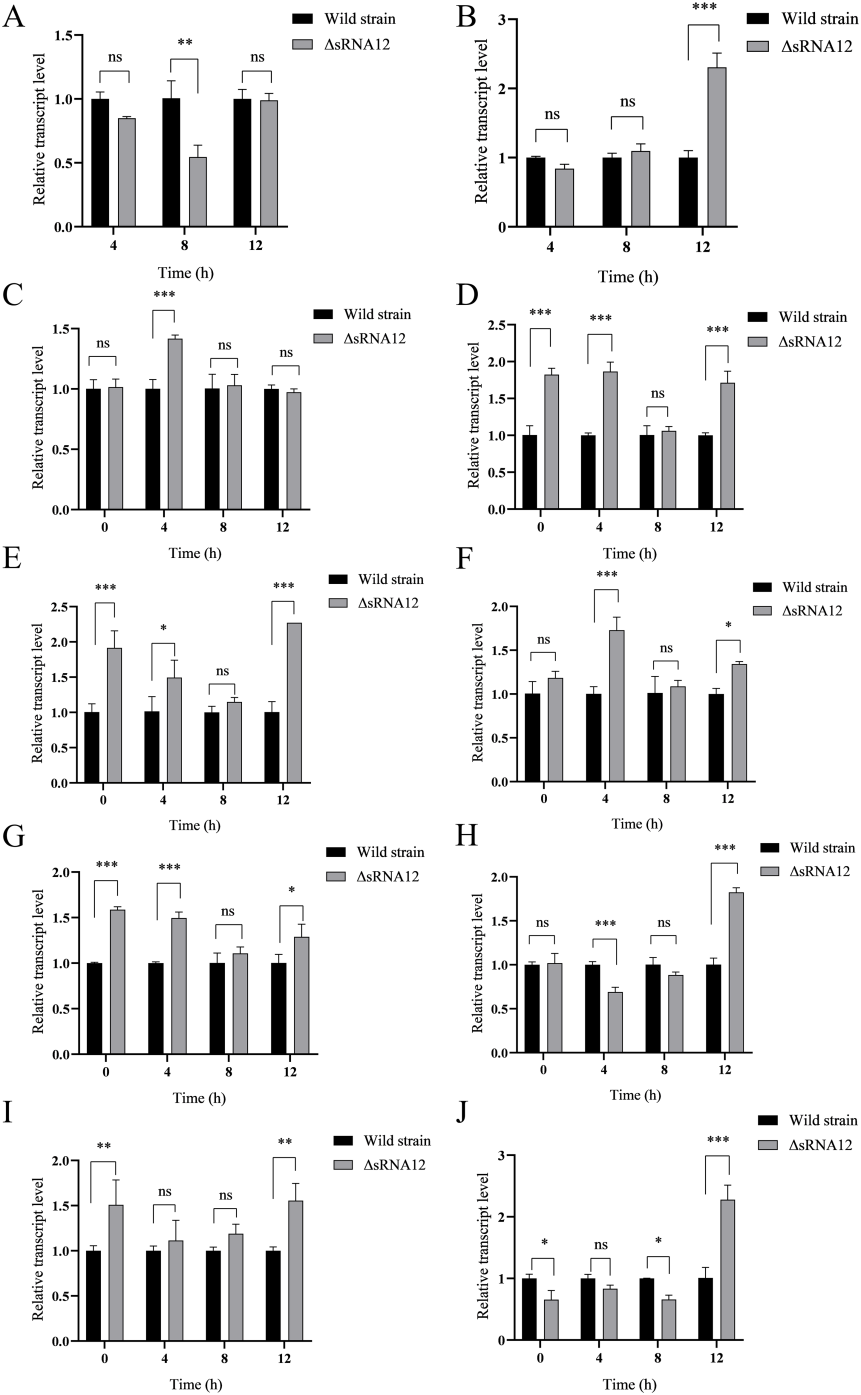
Transcription of target genes within macrophages HD11. **(A)** *bapA*; **(B)** *ompC*; **(C)** *sseC*; **(D)** *ompD*; **(E)** *siiE*; **(F)** *sseG*; **(G)** *prgH*; **(H)** *sseJ*; **(I)** *rho*; **(J)** *fimW.*

The predicted target genes of sRNA12 were ranked according to the minimum free energy, and 10 virulence-related target genes were screened out (Table 2). The levels of the target genes in the cells infected with the wild-type strain and the ΔsRNA12 deletion strain were detected by qRT-PCR. The results showed that compared with the wild-type strain, the expression of *bapA* in the ΔsRNA12 deletion strain was down-regulated at each time point, and it was significantly down-regulated at 8 h. The expression of *ompC* was significantly up-regulated at 12 h. The Ct values of *bapA* and *ompC* at 0 h were>35, so they were meaningless. The expression of *sseC* in the ΔsRNA12 deletion strain was significantly up-regulated at 4 h. The expressions of *ompD*, *siiE*, and *prgH* were significantly up-regulated at 0 h, 4 h, and 12 h. The expression of *sseG* was significantly up-regulated at 4 h and 12 h. The expression of *sseJ* was significantly down-regulated at 4 h but significantly up-regulated at 12 h. The expression of *rho* was up-regulated at each time point, and the differences at 0 h and 12 h were significant. The expression of *fimW* was down-regulated at 0 h, 4 h, and 8 h, and the differences at 0 h and 8 h were significant, while it was significantly up-regulated at 12 h (Figure 4). These findings indicate that sRNA12 can affect the transcription of virulence-related target genes.

**Table 2:** Virulence-related genes regulated by sRNA12.

Gene name	Start site	End site	Free energy (kcal/mol)	Gene function
sseG	311	344	−23.22	Type III secretion system effectors located on SPI-2
bapA	217	262	−22.9	Non-fimbrial adhesin, produced by the type I secretion system, is involved in biofilm formation, bacterial invasion, and adhesion
rho	193	244	−22.77	Transcription termination factor, which can regulate virulence genes
fimW	475	549	−20.61	A member of the type I fimbrial gene cluster, which regulates bacterial adhesion
ompC	726	754	−19.76	Outer membrane protein, which has good immunogenicity
sseC	60	157	−19.15	Type III secretion system translocation protein located on SPI-2
ompD	315	386	−18.93	Outer membrane protein, which has immunogenicity and regulates bacterial adhesion
siiE	1809	1835	−18.82	Non-fimbrial adhesin, encoded by SPI-4, regulates adhesion and invasion
prgH	607	673	−18.63	An important component of the type III secretion system, which promotes the delivery of virulence factors
sseJ	1,059	1,205	−18.57	Type III secretion system effectors of SPI-2

### 3.6 The ΔsRNA12 deletion strain shows enhanced virulence in chicks

To evaluate the pathogenicity of the ΔsRNA12 deletion strain in chicks, the wild-type strain and the ΔsRNA12 deletion strain were orally administered to infect the chicks respectively, and the survival of the chicks was monitored. The analysis showed that the LD_50_ of the wild-type strain (2.11 × 10^1^⁰) was three times higher than that of the ΔsRNA12 deletion strain (7.08 × 10^9^). This indicates that the disruption of sRNA12 significantly enhances the pathogenicity of *S. pullorum* (Table 3).

**Table 3:** LD_50_ of chicks infected with wild-type and ΔsRNA12 deletion strains.

Group	Dose (CFU)	Number of samples	Number of deaths	Mortality rate	LD_50_(CFU)
W1	2 × 10^8^	10	0/10	0%	2.11 × 10^10^
W2	2 × 10^9^	10	1/10	10%	
W3	2 × 10^10^	10	5/10	50%	
Δ1	2 × 10^8^	10	0/10	0%	7.08 × 10^9^
Δ2	2 × 10^9^	10	2/10	20%	
Δ3	2 × 10^10^	10	8/10	80%	
PBS	-	10	0/10	0%	-

### 3.7 Compared with the wild-type strain, the invasion ability of the ΔsRNA12 deletion strain in the visceral tissues of chicks is enhanced

**Figure 5:**
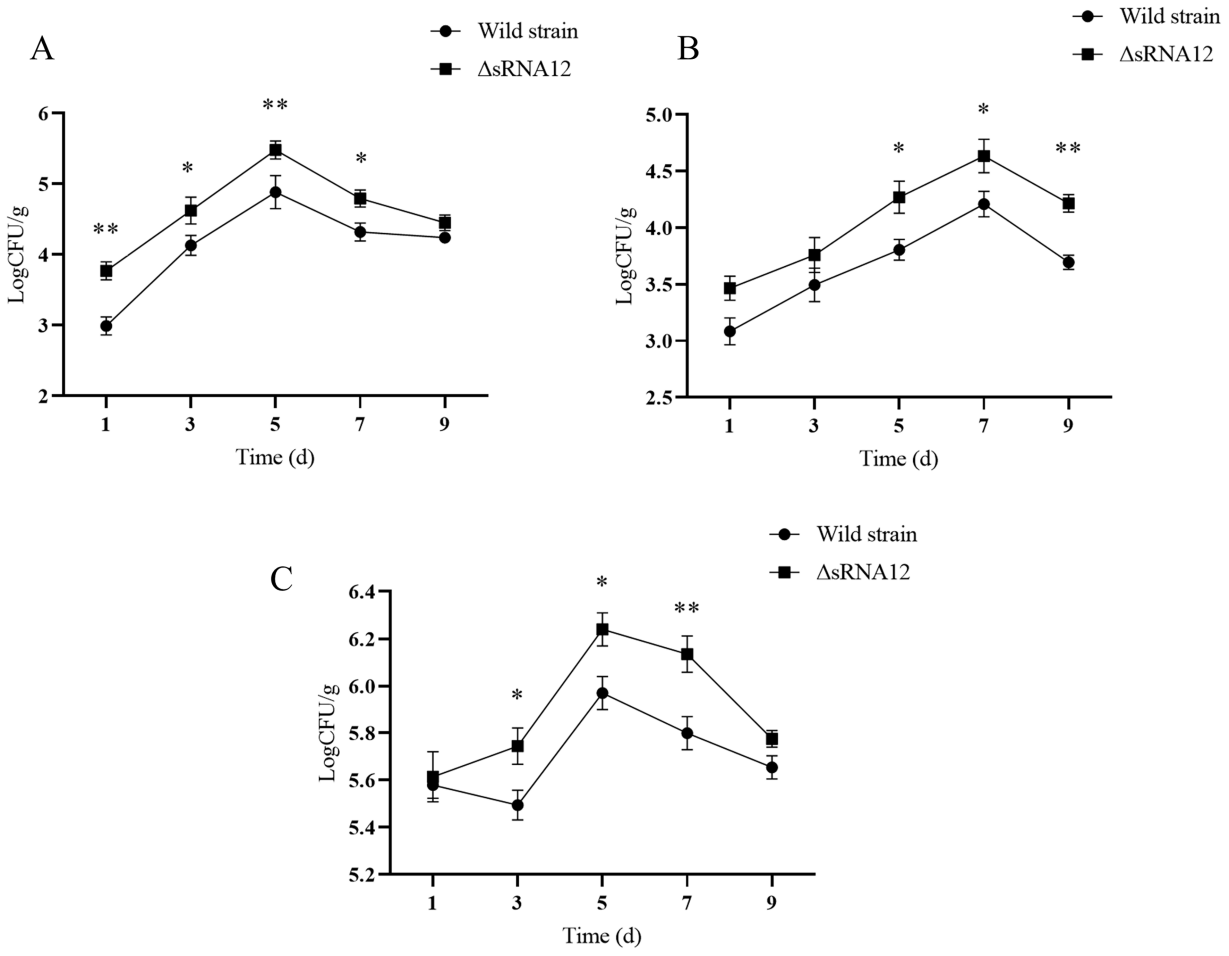
Determination of colonization ability of wild-type and ΔsRNA12 deletion strains in chicks. **(A)** Colonization ability of wild-type and ΔsRNA12 deletion strains in the liver of chicks; **(B)** colonization Ability of wild-type and ΔsRNA12 deletion strains in the spleen of chicks; **(C)** colonization ability of wild-type and ΔsRNA12 deletion strains in the cecum of chicks.

After chicks were infected with the wild-type strain and the ΔsRNA12 deletion strain, we determined the Colony-forming Units (CFU) in the liver, spleen, and cecum. The bacterial colonization in the liver peaked on day 5. In the first 7 days, the bacterial load in the liver of the ΔsRNA12 deletion strain was significantly higher than that of the wild-type strain (*p* < 0.05), with the maximum difference reaching 6.03 times. The colonization number of bacteria in the spleen reached the highest on the 7th day. On the 5th, 7th, and 9th days, the bacterial load in the spleen of the ΔsRNA12 deletion strain was significantly higher than that of the wild-type strain (*p* < 0.05), with the maximum difference reaching 3.31 times. The colonization number of bacteria in the cecum reached the highest on the 5th day. On the 3rd, 5th, and 7th days after infection, the bacterial load in the cecum of the ΔsRNA12 deletion strain was significantly higher than that of the wild-type strain (*p* < 0.05), with the maximum difference reaching 2.16 times(Figure 5). These findings indicate that the deletion of sRNA12 enhances the ability of bacteria to colonize the organs of chicks.

### 3.8 The ΔsRNA12 deletion strain exhibits more severe pathological changes compared to the wild-type strain

**Figure 6:**
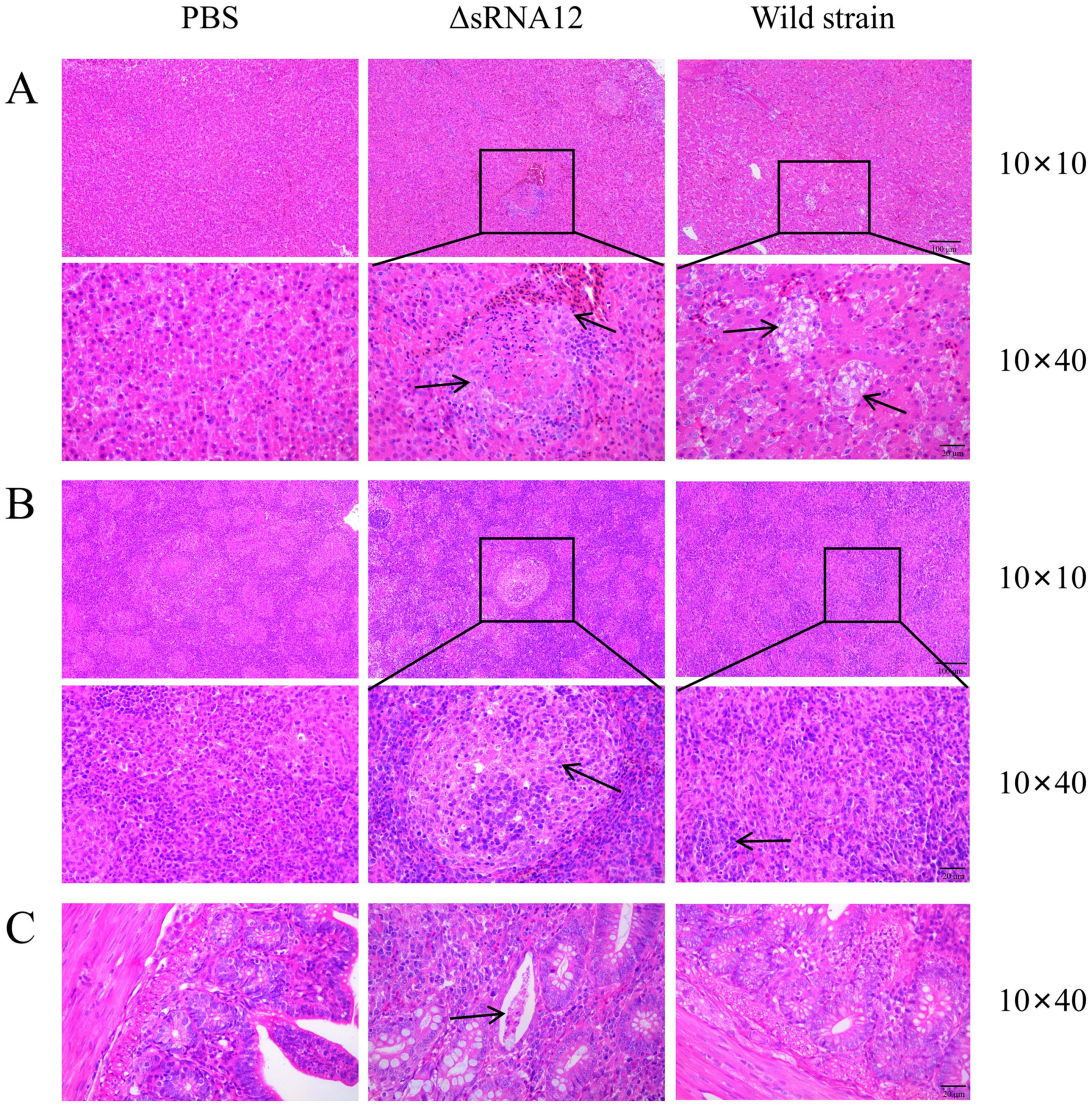
Observation of pathological sections of organs from chicks challenged with the wild-type strain and the ΔsRNA12 deletion strain. **(A)** H&E staining of pathological liver sections from chicks; **(B)** H&E staining of pathological spleen sections from chicks; **(C)** H&E staining of pathological cecum sections from chicks.

H&E staining and pathological analysis were performed on the liver, spleen, and cecum tissues of the infected chicks. The results showed that, compared with the PBS control group, the liver in the ΔsRNA12 deletion strain group showed extensive multifocal necrosis under the microscope. In the necrotic areas, there were infiltrations of inflammatory cells, pyknosis, karyorrhexis, and congestion in the hepatic sinusoids. In the wild-type strain group, focal cellular edema was observed under the microscope, with fewer necrotic foci and less congestion in the hepatic sinusoids, and the overall lesions were relatively milder compared to those in the deletion strain group. For the spleen, in the ΔsRNA12 deletion strain group, multifocal necrosis of splenic nodules was observed under the microscope, and there were infiltrations of inflammatory cells in the necrotic areas. In the wild-type strain group, the boundary between the red pulp and the white pulp was blurred under the microscope, and the splenic nodules were enlarged, with the overall lesions being relatively milder compared to those in the deletion strain group. Regarding the cecum, in the ΔsRNA12 deletion strain group, a reduction in goblet cells in the intestinal crypts, dilation of the intestinal crypts, and mucus containing inflammatory cells and cell debris in the crypts were observed under the microscope. However, there was no significant difference between the wild-type strain group and the PBS control group, and no obvious pathological changes were found (Figure 6). These findings indicate that the deletion of sRNA12 leads to the enhancement of the disease phenotype in the infected chicks.

## 4 Discussion

*Salmonella* is a common foodborne pathogen, and *S. pullorum* is one of the most common pathogens in bacterial infections of poultry. Understanding its pathogenic mechanism is beneficial for better prevention and control of the spread and infection of this disease. In recent years, many small RNAs (sRNAs) have been discovered in bacteria. Similar to microRNAs in eukaryotes, sRNAs are transcribed in the genome but do not encode proteins. They are involved in regulating many physiological processes of bacteria and are important regulatory factors of bacteria. Our previous research preliminarily determined that the sRNA sRNA12 of *S. pullorum* might be involved in the regulation of bacterial virulence through RNA-Seq and bioinformatics analysis. Therefore, in this experiment, we studied the function of sRNA12.

We amplified the full sequence of sRNA12 and obtained a 344 nt fragment, which is consistent with the RNA-Seq results. When chicken macrophage HD11 was infected with *S. pullorum*, it was found that the transcription level of sRNA12 was significantly higher than that of the control group at each infection time point, indicating that sRNA12 may participate in the regulation of bacterial survival within macrophages by targeting the mRNAs of some genes related to bacterial intracellular survival.

We constructed the ΔsRNA12 deletion strain of *S. pullorum* and ensured that the deletion of sRNA12 could be stably inherited, providing a stable sample for subsequent functional identification. The results of the growth rate determination showed that there was no significant difference between the wild-type strain and the ΔsRNA12 deletion strain, indicating that sRNA12 does not affect the growth of bacteria.

In order to successfully establish an infection, *Salmonella* must first resist a series of environmental stresses, adhere to and invade non-phagocytic cells, and finally evade the host immune system to survive within macrophages. Therefore, we conducted environmental stress tests on the two strains and found that the survival rate of the ΔsRNA12 deletion strain decreased in a hyperosmotic environment, increased in a highly oxidative environment, and there was no obvious difference in the survival rate in an acidic environment compared with the wild-type strain. We speculate that sRNA12 may target the mRNAs of certain genes involved in oxidative stress regulation or osmotic pressure regulation, thereby affecting the survival of bacteria under environmental stress.

The cell invasion experiments of the two strains showed that, compared with the wild-type strain, the number of surviving ΔsRNA12 deletion strains within macrophages was significantly higher at each time point after infection. It is speculated that sRNA12 may affect the survival of bacteria within cells through the regulation of the expression of virulence-related genes, the regulation of bacterial metabolism and stress adaptation ability, and the regulation of the interaction between bacteria and macrophages. The specific mechanism needs further exploration.

Subsequently, the target genes of sRNA12 were predicted. The ten virulence-related target genes with the lowest free energy are *bapA*, *ompC*, *sseC*, *ompD*, *siiE*, *sseG*, *prgH*, *sseJ*, *rho*, and *fimW*. Among them, the *sseG*, *sseC*, and *sseJ* genes are located in *Salmonella* pathogenicity island 2 (SPI-2). The proteins encoded by them are one of the effector proteins of the SPI-2 type III secretion system (T3SS), which play a key role in the survival and replication of *Salmonella* within host cells (34). *bapA* is an important gene related to the formation of the biofilm of *Salmonella*. It encodes a large cell surface protein, which is a component of the extracellular biofilm matrix of *Salmonella* (35). In addition, the BapA protein is expressed on the surface of bacteria and secreted by the type I secretion system (T1SS), and can act as an adhesin to mediate the adhesion of *Salmonella* to various surfaces (36). The *rho* gene encodes the Rho protein, which is widely present in prokaryotes and plays a key role in the regulation of gene expression in *Salmonella* (37). *fimW* is a gene closely related to the formation of bacterial fimbriae and is a component of the type I fimbriae of *Salmonella*. The loss of fimbriae will lead to a decrease in the adhesion and invasion of *Salmonella* to epithelial cells (38). *ompC*, *ompD*, and *ompF* encode the three main porins OmpC, OmpD, and OmpF of *Salmonella, respectively.* These porins are immunogenic and can induce an immune response (39). The *siiE* gene is located in *Salmonella* SPI-4. SPI-4 mediates the adhesion of *Salmonella* to the surface of epithelial cells by secreting the SiiE protein, thereby promoting intestinal inflammation (40). The *prgH* gene is located in *Salmonella* SPI-1, and the PrgH protein encoded by it is an important component of the SPI-1 T3SS.

The qRT-PCR experiments of the two strains after invading cells showed that sRNA12 is involved in the regulation of the above-mentioned genes, among which the differences of *ompD*, *siiE*, and *prgH* at each invasion time point are more obvious, and the transcription levels of the three genes in the ΔsRNA12 deletion strain are significantly increased. It is speculated that sRNA12 may participate in the regulation of the virulence of *S. pullorum* by negatively regulating the expression of the above key virulence genes.

Through the challenge experiment on chicks, it was found that the deletion of sRNA12 reduced the LD_50_ of chicks, enhanced the colonization of bacteria in the liver, spleen, and cecum tissues of chicks, and exhibited more severe pathological changes compared with the wild-type strain. Combining the results of the environmental stress test, cell invasion experiment, and target gene regulation test, it is speculated that the ΔsRNA12 deletion strain may enhance the virulence of *S. pullorum* by enhancing the ability to adapt to oxidative stress and promoting the expression of virulence genes, indicating that sRNA12 plays a key role in the host-bacteria interaction process.

## 5 Conclusion

Overall, we successfully predicted a novel sRNA, sRNA12, in *S. pullorum*. The experimental results indicate that the level of sRNA12 significantly increases when the bacteria invade cells. After the deletion of sRNA12, the virulence of the bacteria is remarkably enhanced. This suggests that sRNA12 is largely involved in the process of host-pathogen interaction of the bacteria and plays a crucial role in the regulation of bacterial virulence. Our discovery provides new evidence for the role of sRNA in the regulation of bacterial virulence, offers new insights into the pathogenic mechanism of *Salmonella*, and also lays the foundation for further research on the regulatory network pathways of sRNA, as well as the development of antibacterial drugs and diagnostic tools.

## Data Availability

Original datasets are available in a publicly accessible repository. The raw sequencing data generated in this study have been publicly deposited in the Gene Expression Omnibus (GEO) database. This data can be found here: Accession number GSE269156, https://www.ncbi.nlm.nih.gov/geo/query/acc.cgi?acc=GSE269156.
