# Supplementary figures and images for: A newly discovered sRNA is involved in the virulence regulation of Salmonella pullorum
Source: Front Vet Sci. 2025 Sep 22;12:1651294. doi: 10.3389/fvets.2025.1651294 (PMC12498021; doi:10.3389/fvets.2025.1651294)

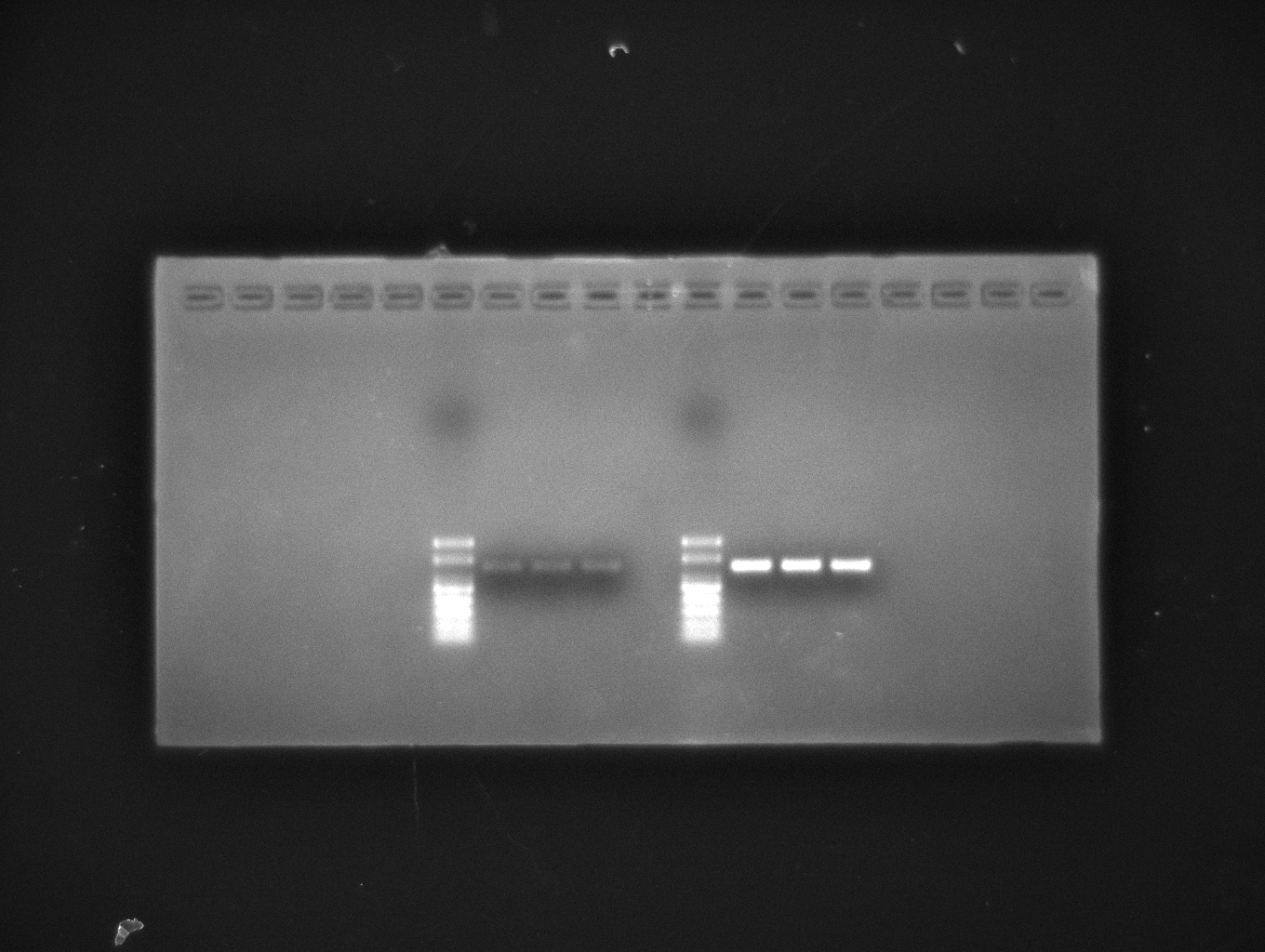

Supplement: Supplementary file 1 [file Data_Sheet_1.zip › Figure S1.tif]

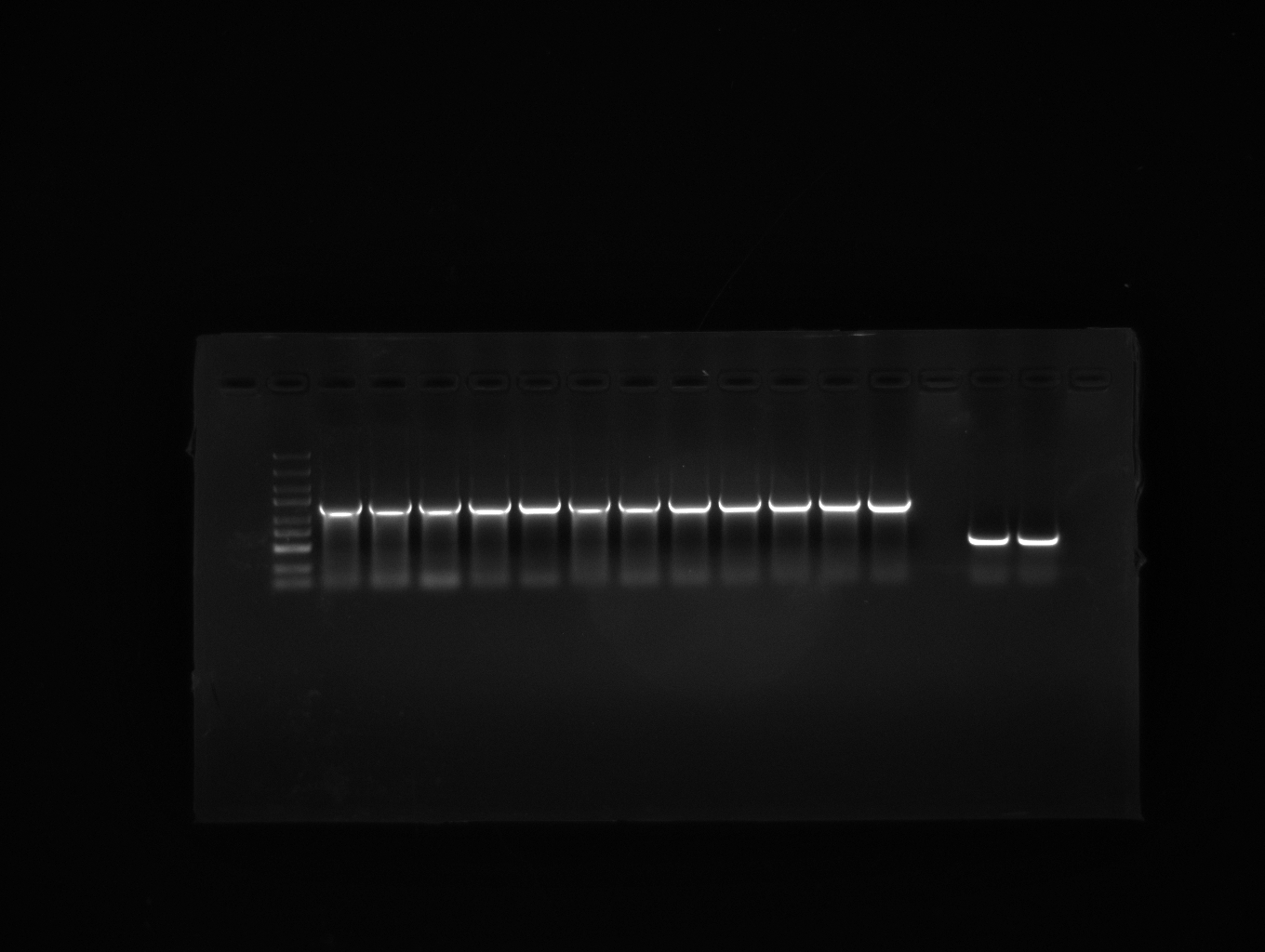

Supplement: Supplementary file 1 [file Data_Sheet_1.zip › Figure S2.tif]

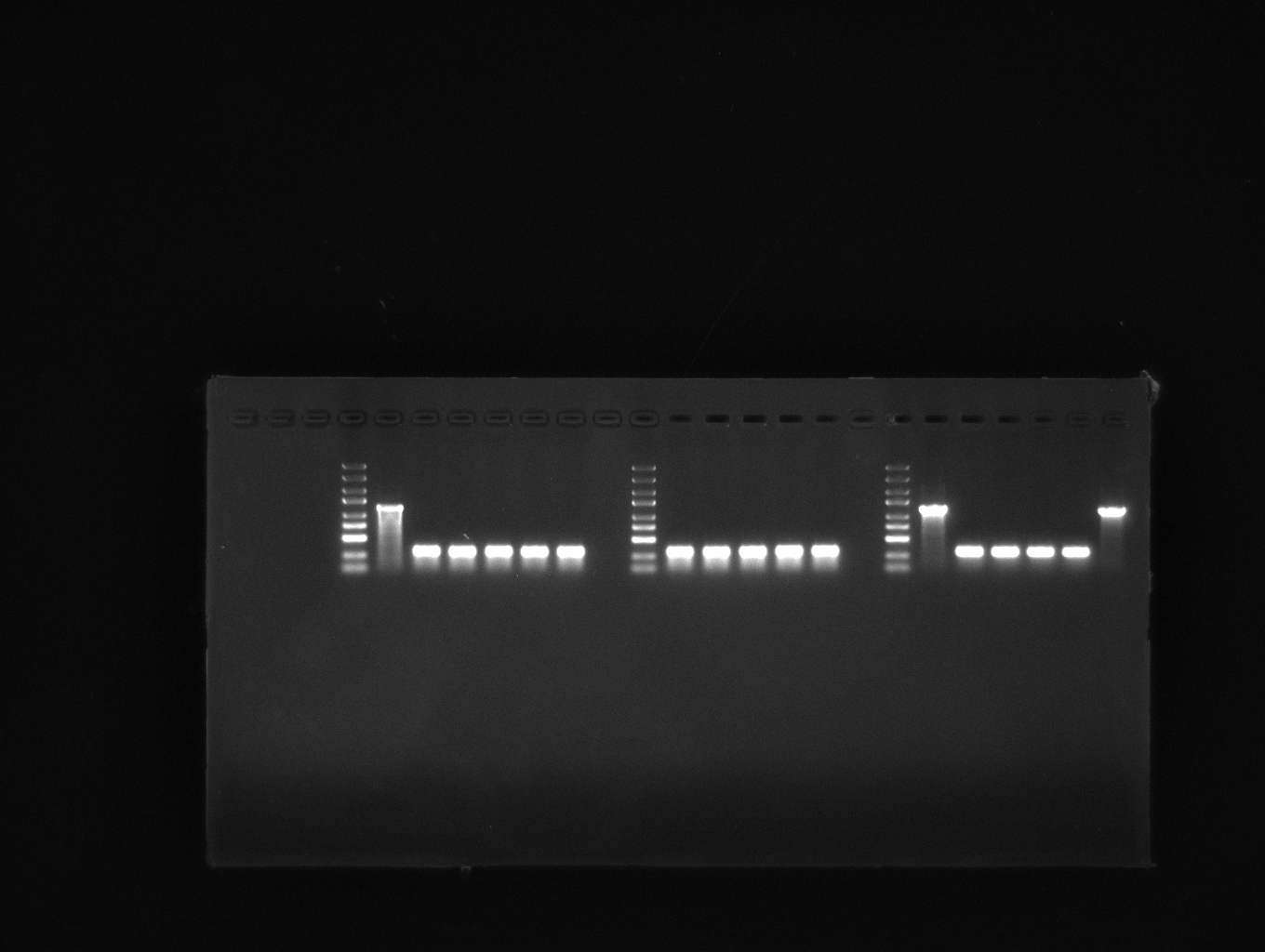

Supplement: Supplementary file 1 [file Data_Sheet_1.zip › Figure S3.tif]
